# Norwegian food system actors’ perspectives on participating in a cross-sector research partnership: a qualitative study

**DOI:** 10.1186/s40795-025-01192-1

**Published:** 2025-12-10

**Authors:** Anne Lene Løvhaug, Lisa Garnweidner-Holme, Laura Terragni, Arnfinn Helleve

**Affiliations:** 1 Institute for Nursing and Health Promotion, Oslo Metropolitan University, Pilestredet 32, Oslo, N-0166 Norway; 2 Centre for Evaluation of Public Health Measures, Norwegian Institute of Public Health, Oslo, Norway

**Keywords:** Cross-sector partnerships, Food profiling, Food systems, Collaboration, Front of pack labelling, Nutrition policy, Stakeholders

## Abstract

**Background:**

Cross-sector partnerships are governance models that are increasingly used in policy, research and practice to address food systems challenges including unhealthy diets. Despite ongoing debates about their value, especially in research, there is limited knowledge on the experiences of participating in these partnerships. This study is set in Norway, a country with tradition for cross-sector collaboration. We examine the Norwegian cross-sector research partnership NewTools to explore participants’ perspectives in an early project stage. The partnership comprises 28 actors from research institutions, food industry, civil society and government agencies, and aims to develop two food profiling models: one for nutrition and one for environmental and social sustainability. This study can help identify initial factors that can influence nutrition-related cross-sector collaboration processes.

**Methods:**

We conducted qualitative interviews with 17 NewTools-participants in 2022. Interviews were analyzed with reflexive thematic analysis.

**Results:**

Five themes were identified: (i) “*The importance of being in the room”* illustrated participants views on the benefits of cross-sector collaboration, including sharing expertise and influencing project outcomes. (ii) “*Divergent expectations towards collaboration and project aims”* reflected varying expectations towards partner involvement and the project outcomes among participants. (iii) “*Viewpoints towards food profiling systems”* highlighted differing viewpoints on two food profiling models to be developed, with more skeptical views towards the nutrition profiling model. (iv) “*Power asymmetries and mistrust”* suggested potential tensions and some preexisting mistrust among partners. (v) “*Pragmatic approach to COI and conflict management”* conveyed that conflicts were considered manageable components of cross-sector collaboration.

**Conclusions:**

In our study from an early project phase, participants described cross-sector collaboration as valuable and challenges as manageable. They also described potential tensions and mistrust between participants and diverging expectations towards roles and project objectives. These findings suggest that potential obstacles for collaboration were present in the project. This underscores the importance of investing time early in cross-sector research partnerships to clarify roles, align expectations and agree on project goals, which otherwise may turn into obstacles for collaboration between actors with varying mandates, interests, and expectations.

**Supplementary Information:**

The online version contains supplementary material available at 10.1186/s40795-025-01192-1.

## Background

Current food systems contribute to unhealthy diets, negative environmental impact, and inequitable conditions in food value chains [1–4]. Improvement of the foods systems requires action from the public and private sector, civil society, and academia [5–7]. One possible approach is through c*ross-sector partnerships*; voluntary, formalized governance arrangements where two or more societal sectors (public, private and/or civil society) collaborate to achieve societal goals [8]. The last decades such partnerships have received increased attention both as local and global governance models [9–13], including in nutrition policy development [11]. A specific variant is cross-sector *research* partnerships, where research institutions are involved as partners. Unlike cross-sector partnerships, whose activities directly impact policy or practice, research partnerships primarily aim to generate new knowledge. Major research funding agencies are increasingly mandating the inclusion of non-research partners in projects in food and nutrition research both at European [14] and national level [15].

Whether in policy, practice or research, cross-sector partnerships may have several advantages, including leveraging expertise to address complex challenges; pooling financial resources; building consensus; enhancing policy support and contributing to feasible outcomes [9, 11, 16]. However, such partnerships may also have challenges. Collaboration between actors with varied mandates, interests and values can lead to tensions and mistrust [11, 17], and powerful actors may influence processes and outcomes towards their own interests, potentially leading to conflicts of interest (COI) at the expense of public health [18–20]. In particular, literature has problematized the role of the commercial food sector in partnerships due to its role towards unhealthy food environments, which may not be aligned with objectives for improving food quality and diets [21, 22]. This debate also centers around food industry involvement in nutrition research which can compromise research integrity, especially when research is related to policy [20, 22].

While several studies have assessed the effectiveness of cross-sector partnerships on public health [23], relatively few studies have explored how participants in nutrition-related cross-sector partnerships experience collaboration. Studies from the United Kingdom [24, 25], the Netherlands [9] as well as studies investigating international partnerships [17, 26] have indicated that power asymmetries, trust deficiencies and conflicting interests among partners constrained collaboration processes.

This study focuses on “NewTools”, a Norwegian cross-sector research partnership co-funded by the Norwegian Research Council [27]. Norway has a tradition of habitual engagement between governments and other stakeholders in nutrition policy development. For example, the Nordic Keyhole (a voluntary, government-owned front-of pack nutrition label) was developed through consultations with all sectors [28]. Furthermore, a public-private partnership between health authorities and food industry has been established to facilitate healthier population nutrition [29]. Even if cross-sector (research) partnerships are welcomed and supported, less is known about the experiences from institutions that are involved.

The overall purpose of NewTools is to develop two food profiling models – one for nutritional quality and another for sustainability (i.e., environmental and social impact) through a cross-sector research partnership. Food profiling models enable assessment and ranking of foods and beverages [30] and can underpin a range of policy actions like school food standards, regulation of nutrition and health claims, and front of pack labelling [31]. In NewTools, the food profiling model for nutrition builds on the existing Nutri-Score [32] algorithm, whereas the profiling model for sustainability is developed more from scratch. In NewTools, project partners from different parts of the food system are expected to provide perspectives which can be considered in the development of the food profiling systems. They are also invited to explore possible applications [27].

Conceptually, literature states that conditions present at the early stages of a partnership—such as preexisting relations, the level of trust between participants, distribution of power, and expectations towards collaboration and outcomes—will influence collaboration processes and results [13, 33]. Exploring the perspectives of partners representing different interests can shed light on these conditions and help understand the potential and challenges in cross-sector research partnerships. Given that the NewTools project addresses contested, policy-related issues, it offers a unique opportunity to explore both the benefits and obstacles of cross-sector partnerships.

The aim of this study was to explore NewTools-partners’ initial perspectives around project participation, focusing on expectations towards participation; viewpoints on food profiling systems; and reflections around COI and stakeholder management.

## Methods

This study uses a qualitative design. We conducted qualitative individual interviews with NewTools participants representing different societal sectors and interests to explore their perspectives during an early project phase. The study took place five months into the project (June 2022), when only a few project activities had taken place. This included a digital kick-off meeting and a physical consortium meeting. Partners had also been invited to provide written input to the development of a framework for engagement (described further below), however, they had not yet been involved in work with the food profiling models.

### Study setting and participants

The NewTools project runs from 2021 to 2025. It is led by the Norwegian Institute of Public Health, which is a government agency under the Ministry of Health and Care Services but also an independent research institution. The leadership role in NewTools is in the latter capacity. In total the project involves 28 formal partner organizations representing (i) research institutions, (ii) food industry, (iii) civil society, and (iv) government agencies (Table 1).

**Table 1:** NewTools partners

Sector	Partner organization
Research institutions	Norwegian Institute of Public Health^a^
	University of Oslo^a^
	Norwegian University of Life Sciences^a^
	Norwegian Institute for Sustainability Research^a^
	Statistics Norway^a^
	Oslo Metropolitan University – OsloMet^a^
	Institute of Marine Research
Food industry	Nortura (egg and meat producer)^a^
	TINE (dairy producer and manufacturer)^a^
	Felleskjøpet (cooperative for grain and farmer operating assets)^a^
	Norgesmøllene (grain, flour and baked goods manufacturer)^a^
	Animalia (meat and poultry research centre)^a^
	BAMA (fruit and vegetable importer and manufacturer)^a^
	NorgesGruppen (retail)
	REMA 1000 (retail)
	Coop (retail)
	Orkla (manufacturer)
	FoodDrinkNorway (federation for food-, drink and bio industry)
	Norwegian Farmers’ and Small Farmers’ Association
	The Norwegian Seafood Federation
Civil society	The Norwegian Consumer Council^a^
	Future in Our Hands (environmental organization)
	EAT Foundation
	Spire (youth environmental and development organization)
	Matvalget (counceling service for food service operators)
Government agencies	Norwegian Directorate of Health
	Norwegian Food Safety Authority
	Norwegian Agriculture Agency

^a^*Collaborating partners* that cover costs of own activities and report time spent to the Research Council. All other organizations are *associated partners* that cover costs of own activities but are not obligated to report time spent

All partners are formally committed to the project through having signed a consortium contract. Six research partners and one civil society partner receive funding from the Norwegian Research Council. All other partners cover the cost of their own activities in the project, however, six partners representing food industry are also obligated to report time spent on project activities to the Research Council which counts towards the project’s budget. No money flows from these partners to research institutions. All non-research partners were invited to the project based on having sustainability and/or nutrition experience and expertise, and relevant roles in the Norwegian food system. In addition to the consortium contract that formally governs the project, a framework for engagement has been developed that concretizes partners’ roles and responsibilities [34]. Researchers representing research institutions lead research activities, involve partners, and make decisions. Three of this study’s authors (ALL, AH and LT) are employed at academic partners in NewTools.

### Recruitment

We undertook recruitment in May 2022 based on a purposive sampling strategy aiming to recruit at least 12 NewTools project participants with representation of each of the four sectors involved. All NewTools contact persons from food industry, civil society and government agencies, and three contact persons from the research institutions were invited by e-mail. After one week we sent reminders to contact persons representing government agencies as this partner group was lacking representation. In total, 17 out of 24 invited contact persons accepted to participate. Five food industry partners did not respond (reason unknown), one food industry partner had to cancel a planned interview due to other obligations, and one government partner declined due to limited capacity. Ahead of the interviews the participants received information sheets (Additional file 1) and submitted written informed consent.

### Data collection

We developed a semi-structured interview guide including three main topics: (1) expectations to the project, (2) viewpoints on food profiling systems, and (3) reflections around collaborations between different societal sectors, COI, and stakeholder management (Additional file 2). The topics were chosen based on literature focusing on COI in cross-sector partnerships and conceptual literature [13]. The interview guide was pilot tested for clarity and relevance with one participant representing civil society. This did not lead to changes, and the interview was included in the analysis. The first author (ALL) conducted the interviews. ALL is a public health nutritionist and PhD candidate with experience in qualitative research and with an interest in the role of governments and the private sector towards healthy food environments and nutrition policy. She has previously worked with policy advocacy regarding restrictions on food marketing towards children. The co-authors are experienced qualitative researchers and have broad experience with nutrition policy research. Through her role in NewTools, ALL had met most of the participants ahead of the interviews. This included the participants representing the research institutions, which she had met and worked with regularly from the project start. ALL also knew a few of the participants from other professional contexts. This meant that there were varying preexisting relationships between the researcher and participants. All participants received the same background information and were asked the same questions (Additional file 2).

Twelve interviews were conducted digitally (Zoom) and five in person. Interviews lasted between 30 and 60 min. They started with an introduction which included a description of public health nutrition literature focusing on COI as a background of the study, the role of the interviewer, and data protection measures. Participants were asked to express the views of their organizations and informed that their input would be linked to the sector they represented. All interviews were digitally audio recorded and manually transcribed verbatim.

### Data analysis

ALL analyzed the interviews guided by Braun and Clarkes’ reflexive thematic analysis [35], involving the following iterative phases: familiarization involved listening to audio recordings, note-making of overall impressions, and transcription. Audio recordings and transcripts were accessible for the research team only. This was followed by the formal analysis process, facilitated by NVivo qualitative data analysis software (QSR International). All interviews were coded by ALL, guided by the research questions and keeping close to the interview data. The co-authors read 2–5 interviews each and discussed the coding with the main author to ensure methodological rigor. They remained involved throughout the analysis process, as described below. In an iterative process, ALL grouped codes with similar content into tentative themes at a more interpretive level. These themes were revised and refined through several iterations, which included discussions of the coding tree with co-authors; visualizing relations between codes and themes; several rounds of interpretations discussions considering the interviews; and writing memos about code and theme development. Lastly, during the writing up process, themes were given names and defined [35]. Overall, the process of theme development was inductive, based on patterns in the data.

To ensure that quotes accurately represented participants’ viewpoints, we sent the quotes and Norwegian transcripts to 13 quoted participants before submitting the manuscript. This resulted in the following changes: (1) removing a quote that did not represent the official position of the partner organization; (2) amending the wording in two quotes for more nuanced translations; and (3) adding a sentence that had originally been omitted from a quote.

## Results

### Participants

Seventeen representatives from project partners in NewTools were interviewed about their initial perspectives on project participation. Participants from *government agencies* (*n* = 2) represented health authorities. The majority of *food industry* partners (*n* = 7) represented large organizations involved with food production or manufacturing. Five participants represented relatively smaller *civil society* organizations were three primarily focused on environmental and social sustainability and two also addressed nutrition and health. Three participants represented two different *research institutions* which specialized in nutrition/health and sustainability, respectively. Around two thirds of the participants were women and most participants had relatively senior positions in their organizations.

### The importance of being in the room

Participants were asked why they had decided to take part in NewTools and reflected around pros and cons of cross-sector collaboration. Across the sectors involved, participants described collaboration and the inclusion of actors having different roles in the food system as an important element of NewTools. Many participants mentioned that, due to the complexity of the project’s aims, inclusion of different perspectives and actors would benefit project outcomes and increase the likelihood that project results were feasible and applicable. As explained by one civil society participant:

*“If it had only been academic and one hadn’t included those who might use the labelling scheme on their products*,* one would actually have missed doing something that could have an impact out there.”* (Interview 1, civil society).

Several participants described NewTools as an opportunity to share their expertise, discussing how NewTools was directly relevant for the mandates of their organizations and that their experience and expertise could benefit the project. For some participants, participation in the project was seen as an opportunity to speak their interests. One participant explained:

*“It* [participation in the project] *clearly aligns with the project’s purpose*,* which is to develop a scoring system for both sustainability and health. (…) Front of Pack nutrition and sustainability labelling are significant to our members because they impact the products they produce*,* what they can sell*,* and how they label their products*,* which in turn affects their competitiveness. (…) We have a mandate to engage in topics that concern our members. Therefore*,* it was evident that we needed to allocate resources to this.”* (Interview 17, food industry).

Some project participants appeared to have a broader motivation beyond contributing to research. Participants commonly referred to the European Union Commissions’ 2020 Farm to Fork strategy, which included proposals for a harmonized front-of-pack nutrition label (FOPNL) and a sustainable food labelling framework [36]. Thus, NewTools appeared as a relevant venue for being informed about and potentially being engaged in relevant EU policy processes. The link between NewTools and EU policies was described by one participant:

*“There’s a lot happening now with labeling in general*,* so. That’s of course why we are participating in this as well*,* so that we can keep track of what’s happening*,* and so that we can also contribute a bit to what’s happening in the EU. What happens in the EU feels very distant from us*,* but here we can get a bit closer.”* (Interview 7, food industry).

Participants seemingly considered that collaboration and representation from many sectors enhanced the relevance and legitimacy of NewTools. Project participation could stem from altruistic but also strategic motivations, including protecting interests in the context of “real world” policy development.

### Divergent expectations towards collaboration and project aims

Participants from the different sectors expressed different expectations towards the roles of partners and the nature of the project aims. Participants representing civil society and food industry described an expectation that the food profiling systems should be developed through their direct involvement and consensus-based decision making. For example, participants said:

*“For us*,* it’s important to be involved now and lay the foundation for the scoring systems. If we can only nitpick at the end*,* it’s not so relevant to participate.”* (Interview 9, Civil society).

*“I think it is very important that we agree on a system and that we agree on the indicators that should be included in a scoring matrix.”* (Interview 5, food industry).

This expectation appeared to contrast with that of project researchers. Researchers described the planned engagement of non-research partners as consultation, not consensus-building, suggesting a more distanced role for partners. As noted by one researcher:

*“I believe some actors still think that this is a project where we should come to an agreement on things*,* despite the governing documents are quite clear that we are not seeking agreement*,* but we are seeking discussion*,* to get all perspectives on the table. I think it takes some time to fully understand and accept this approach.”* (Interview 2, research institution).

Expectations also varied regarding project aims, i.e., the food profiling models to be developed. While these models can have different applications, they are closely related to front of pack labelling [31]. In the interviews participants often equated food profiling models with front of pack labelling policy, as also described in the theme above. This may suggest that NewTools could be understood not only as a research project but also as policy development.

Participants’ expectations to whether project outcomes would be ready for implementation at the end of project also varied. While some participants representing civil society and food industry expected results that were ready for implementation, researchers had more modest expectations. This discrepancy is illustrated below.

*“To what extent do I imagine…? It’s the whole purpose of the project*,* so I envision that we must get there*,* otherwise the project won’t be successful!”* (Interview 5, food industry).

*“(…) I don’t have any expectation that there should be … that there should be a ready-made*,* push-the-button system or framework when we finish. I don’t believe that. The reason I don’t believe that is because it’s so complex. It requires a lot of maturing*,* and we will probably need many rounds of deep discussion*,* before we think we have a good proposal.”* (Interview 14, research institution).

The apparently divergent expectations among participants suggested a level of uncertainty regarding the roles of partners, distribution of power, and the overall purpose of the project.

### Viewpoints towards food profiling systems

Expectations towards the outcomes of a partnership has been described as a driver influencing motivation to participate in collaborative initiatives [13, 33]. Differences in expectations became clearer when discussing the food profiling models for nutrition and for sustainability to be developed.

Participants’ viewpoints towards the two models appeared to differ along several factors. Viewpoints on the planned profiling model for sustainability seemed to be rooted in overarching, broad perspectives. Several participants across sectors emphasized *complexity*; the sustainability food profiling system needed to go beyond climate and include social and economic sustainability dimensions, as noted here:

*“(…) we see that sustainability is so much more than that [climate]*,* and it might become even more important in the future. The working environment and these risk ingredients coming from countries where working conditions might not be so good.”* (interview 7, food industry).

Other participants, representing both food industry and civil society, emphasized the value of *objectivity*, arguing that indicators for measuring aspects of sustainability should be independent from Norwegian or other vested interests. This could enable improvement of food industry practices. One participant reflected:

*“At the kick-off meeting we had a discussion where someone stated that ‘we must make sure that the Norwegian farmers come out well from this’ and then I was quite upset inside. Because that’s not what this project is about*,* this project should reflect the* *truth*, *and then we must rather adapt. I think that’s quite important.”* (Interview 5, food industry).

One participant highlighted *alignment* between the food profiling model and existing environmental impact methodologies. In contrast to participants who emphasized complexity when considering sustainability, this participant considered that adding additional sustainability dimensions was problematic:

*“In the PEF* [Product Environmental Footprint] *method*,* you calculate the size of 16 footprints. (…) If you’re up to that*,* it’s complicated enough. If on top of that*,* you’re going to have some social stuff*,* you can’t merge it all.”* (Interview 12, food industry).

In contrast, viewpoints on the planned profiling model for nutrition were mainly related to FOPNL and specifically Nutri-Score, whose algorithm was used for further development in NewTools. Consequently, reflections tended to be more technical, often addressing perceived flaws in the algorithm. Reflections also showed that many partner organizations already had established policy positions towards Nutri-Score.

Only a few civil society participants were positive towards Nutri-Score, as expressed here:

*“I think it is very positive. My workplace has wished for this for a long time*,* thinking that the Keyhole label* [the existing FOPNL in Norway] *has been helpful for many consumers*,* but we also see that it has some weaknesses. We also think that it could be good to label unhealthy food in some way.”* (Interview 1, civil society).

However, most participants, across sectors, criticized Nutri-Score for algorithm weaknesses, leading to flawed scores and consumer confusion.

*“The scores given to different foods is problematic. (…) Full-sugar jam gets a better rating than mackerel in tomato sauce. But one could argue that for the general Norwegian population*,* it’s more beneficial to eat mackerel in tomato sauce than jam.”* (Interview 7, food industry).

Criticism towards Nutri-Score often concerned political issues. Participants from government agencies and food industry emphasized discrepancies between Nutri-Score and existing Norwegian nutrition policies including the Keyhole. The Keyhole was seen as better aligned with dietary guidelines, well-known, and supported by the industry. Its voluntary nature was also valued, as one participant from the food industry noted.

*“(…) we think we have a well-established label in Norway*,* which is the Keyhole. It has required* *a lot* *from both the authorities and the industry*,* in collaboration*,* to develop it. And the industry has really embraced it. And it’s a voluntary label. So*,* it depends a bit on whether the European one becomes mandatory or voluntary.”* (Interview 11, Food industry).

Whereas participants’ viewpoints towards the two project objectives represented different perspectives, viewpoints towards the nutrition profiling system appeared as more skeptical and politicized given the relation between nutrition profiling and the FOPNL Nutri-Score.

### Power asymmetries and mistrust

Some participants highlighted a concern for potential power asymmetries as potential challenges for the project, and certain pre-existing tensions and mistrust – associated with partners’ varying mandates and interests – appeared to be present.

A couple of the civil society participants expressed concerns that more powerful project partners could dominate the project. Specifically, this applied to food industry partners contributing with in-kind funding to NewTools [27], which were considered as having more time and financial resources to invest in project participation. Their roles towards financing appeared to amplify these perceived asymmetries.

*“(...) a concern we perhaps have*,* is that there is very different power relations within the partners involved. Some contribute with a lot of funding and have a completely different control over how the project will proceed*,* while we’re just supposed to contribute with ideas and such*,* we do not have any financial contribution to the project (...) beyond labor. So that (...) is a concern*,* how the power relations will be between the different participants.* (Interview 9, civil society)

Some participants from the food industry, government and civil society described preexisting tensions between food systems actors beyond the NewTools project. Such tensions could stem from misalignment between stakeholders’ different mandates and objectives. They could also stem from disagreements between sectors and disciplines over the legitimacy and ownership of a field. One participant described tensions between the agriculture and nutrition fields:

*“I think perhaps that agriculture may experience … when the nutrition field starts working with sustainability*,* and they get quite a lot of resources to it*,* and starts to manage and arrange… Maybe*,* for some of them*,* there are some linkages* [in agriculture] *they’re not fully aware of.”* (Interview 13, Food industry”).

Mistrust among partner groups may be a source of tension in cross-sector partnerships [13, 17]. Some participants from the food industry described that other nutrition stakeholders, including nutrition researchers, mistrusted food industry actors, as exemplified here:

*“I know of researchers who have left academia for industry and were almost called traitors*,* right. (…) But I think that might be starting to change a bit*,* it was perhaps (…) more like that before (interview 11*,* food industry)*.

### Pragmatic approach to COI and conflict management

Participants identified a range of potential project challenges, from technical disagreements to conflicting interests and values. The latter were often termed goal conflicts or conflicting interests. Only a few participants from research institutions and civil society raised (financial) COI as a challenge without prompts from the interviewer. Participants across sectors described potential conflicts including COIs as inevitable and manageable elements of cross-sector collaboration. A participant from the food industry explained the importance of considering conflicting interests:

*“One can sit in academia and believe that the knowledge one generates can somehow change the world. Then you forget all the conflicting interests and cross-considerations that must be considered simultaneously. (…) While the private sector (…) constantly have to connect all the cross-considerations coming from various public bodies*,* input from academia*,* all more or less self-appointed experts in the media.”* (Interview 12, Food industry).

Suggested strategies for managing conflicts and COI included active partner involvement, transparency, and effective communication. The importance of involving partners can be linked with partners’ expectations towards collaboration and contributing to the project as described above, and as noted by this participant:

*““The most important thing for us is to be included in the rounds that occur in the project*,* so that we can provide input. (…) we also want the scientific aspects to determine the outcome. (…) It is crucial that we have the opportunity to contribute during the consultation rounds*,* and that things do not happen behind closed doors*,* resulting in a draft that is almost fully established.”* (Interview 16, Food industry).

In addition, some participants from civil society and food industry emphasized the importance of a project’s composition in preventing COI, as projects with less stakeholder groups could pose risks to reputation and create COIs. They considered NewTools less risky due to representation of many sectors and interests and credible research institution leadership:

*“We are quite cautious about entering into industry collaborations where it’s just the two of us without a research institution present. Because we perceive there is a risk that we will be pushed in front*,* in a way … so that an industry actor can say okay*,* look here we have a partnership with this organization*,* thereby we get a sort of sustainability stamp.”* (Interview 8, Civil society).

Finally, one single participant raised that the project should agree on a common problem understanding:

*“A scoring system is*,* in a way*,* a tool to solve a challenge. But then you kind of go straight to the tool*,* without having a … (…) people may have completely different perceptions of what the challenge is. I wish we had spent more time discussing that.” (Interview 13*,* Food industry)*.

## Discussion

### Summary of key findings

This study examined the initial perspectives of participants representing diverse societal sectors engaged in a cross-sector research partnership aiming to develop food profiling models for nutritional quality and environmental and social sustainability. Participants described cross-sector collaboration as beneficial for the project. The findings indicate that although expectations regarding participation varied, there was a shared perception of the partnership as a potential arena for exerting influence and promoting interests. Notably, participants expressed stronger, and often more skeptical, views concerning the nutrition profiling model compared to the sustainability profiling model.

Moreover, they identified a range of potential challenges associated with involvement in the partnership, including conflicting interests and relational tensions stemming from partners’ interests and mandates. These challenges were regarded as inevitable yet manageable aspects of collaboration, with active partner involvement serving as an important preventive strategy.

### Results discussion

The main criticism against nutrition-related cross-sector partnerships is the risk that COIs, in particular financial interests, can undermine processes and outcomes [18–20]. In the NewTools project, COIs are clearly present as several project partners have financial interests that could be affected by food profiling models and potential policy measures based on these. Furthermore, study findings indicated that project participation was seen as a relevant arena for protecting interests. The perceived policy relevance might be influenced by the presence of government agencies in the project. Hence, one motivation for NewTools participation could be to influence front of pack labelling policy development, in particular related to nutrition labelling. Despite this, COIs did not come across as a major concern in our study, where the value of collaboration to address food systems challenges appeared to outweigh potential challenges. Although participants from research institutions and civil society seemed to be more conscious of challenges related to COI, participants across sectors described COI and other conflicts as manageable through relatively standard project steering strategies. This perspective is similar to a “multistakeholder” approach to cross-sector partnerships where these are seen as arenas for discussion, knowledge-sharing and negotiations that assumingly can contribute to a common understanding of a problem, underpinning acceptable and feasible outcomes [11, 13, 33]. Our findings may reflect the Norwegian context and a so-called “Nordic model” where stakeholders are accustomed to cross-sectoral engagement, negotiations and compromising due to a history of active involvement of stakeholder groups in policy development processes [37].

A hallmark of the Nordic model is the high level of trust among groups of societal stakeholders [37], which facilitates cross-sector collaboration [13]. In contrast, a lack of trust can present significant challenges for collaboration [13, 17]. In this study, a presence of trust could potentially explain the seemingly positive attitudes towards cross-sector collaboration and the limited concerns about challenges such as COIs. This would align with findings from an evaluation of the Norwegian public-private partnership for a healthier diet, which highlighted trust as a key foundation for collaboration [38]. However, our findings indicated some preexisting tensions and signs of mistrust among partner groups, suggesting that the narrative of trust in Nordic countries [37] may not apply equally to all stakeholder groups or contexts. The mistrust observed in our study likely stemmed from differing mandates and interests among stakeholders, including the commercial interests of the food industry. Both international and Norwegian studies have argued that real or perceived COIs involving the food industry can undermine trust among food system actors engaged in cross-sector partnerships [17, 39]. Because initial low levels of trust in cross-sector partnerships can lead to reduced commitment [13], literature recommends investing time in trust-building strategies, for example through developing clear ground rules, defining roles, and ensuring that all stakeholders are empowered to contribute [13].

Our study findings suggested that researchers and other participant groups had different expectations towards partner involvement in the development of food profiling systems. In line with recommendations for nutrition-related cross-sector partnerships [22], including food profiling model development [40], decision-making authority on the profiling models in NewTools has been allocated to project researchers. Whereas this may contribute to managing COIs and safeguarding research integrity, this power distribution model could imply a more distanced role for project partners than some expected at this initial project phase. This could reduce partner’s motivation for project participation, as literature posits that motivation to engage in cross-sector collaborations increases when participation is considered concrete and tangible but decreases when it is not [13]. Hence, recommended COI management measures may become an obstacle for collaboration in cross-sector partnerships. This underscores the importance of clear communication about roles and responsibilities in the early stages of cross-sector partnerships.

Finally, an important enabler of cross-sector partnerships is to have shared goals or a “shared theory of change” which involves stakeholders agreeing on the scale of a problem and the means for addressing it [13, 17, 33]. In our study, it appeared that participants had different understandings of the projects’ purpose and aims, such as whether the project was intended to generate knowledge or develop policy through front of pack labelling. Furthermore, several participants were critical of the Nutri-Score algorithm, which formed the basis for the work on the nutrition profiling system in NewTools. This suggests that at this early phase, shared goals had not been established, indicating a potential for tensions in the project going forward. Indeed, literature has argued that a lack of shared goals can lead to participants withdrawing from partnerships [33], and that developing shared goals between stakeholders with widely differing mandates and interests is a critical achievement requiring time-demanding processes including face-to-face dialogue and repeated interactions between participants [33].

### Strengths and limitations

Strengths of our study include the inclusion of seventeen participants from four different societal sectors involved in a concrete cross-sector research partnership. The qualitative interviews enabled exploration of different perspectives and reflections in an early project phase. This may help identify factors that could influence the collaboration process going forwards. Respondent validation ensured that quotes represented the viewpoints of participants’ organizations.

Our study also has limitations. We interviewed “insiders” in a voluntary research partnership, whose opinions towards cross-sector collaboration may differ from other stakeholders. Further studies that include perspectives of external stakeholders would be valuable. Also, results from our study are likely not transferable to all cross-sector partnerships but could have relevance for similar contexts. We also acknowledge that the data collected in qualitative interviews are influenced by the researchers. The background of our study was literature and debates around cross-sector partnerships in nutrition, in particular literature focusing on COI [18–20]. To balance this relatively critical perspective, we have also looked to partnership literature from other disciplines [13, 33]. Furthermore, the varying preexisting relationships and possible power relations between interviewer and participants may have influenced the interviews. However, we cannot be sure in what way this may have occurred [41]. Finally, since there has been a time lag since the interviews were conducted, later project experiences may have influenced our analysis. We will conduct new interviews to explore experiences gained in the NewTools project, which will complement this study.

## Conclusion

This study adds to the empirical research on cross-sector research partnerships in food systems drawing on participants’ perspectives. Focusing on the early phases of a partnership enables identification of factors that may influence the further collaboration processes. Our study findings may help understand benefits and challenges for cross-sector collaboration, in particular in contexts with traditions for engagement and partnership between different societal sectors. In an early project phase, study participants considered that collaboration among different food systems actors would benefit the project and that potential challenges could be managed through project steering. Possible tensions and mistrust between participants and diverging expectations towards roles and project objectives also emerged, suggesting that potential obstacles for collaboration were present in the project. Our findings underscore the importance of investing time early in cross-sector partnerships to clarify roles and responsibilities, align expectations and agree on project goals.

## Supplementary Information

Supplementary Material 1.

Supplementary Material 2.

## Data Availability

The datasets used and/or analyzed during the current study are available from the corresponding author on reasonable request.
