# Supplementary material for: Norwegian food system actors’ perspectives on participating in a cross-sector research partnership: a qualitative study
Source: BMC Nutr. 2025 Dec 10;12:4. doi: 10.1186/s40795-025-01192-1 (PMC12784486; doi:10.1186/s40795-025-01192-1)
Supplement: Supplementary file 1 — Supplementary Material 1. [file 40795_2025_1192_MOESM1_ESM.docx]

*This information sheet has been translated from Norwegian to English for the purposes of this article.*

Would you like to share your expectations and experiences with participating in NewTools?

We invite you to participate in an interview where the aim is to investigate expectations and experiences with participating in NewTools as an interdisciplinary research project within the Norwegian food system. The interviews can help facilitate the process in NewTools and ensure that all parties are involved in a constructive manner. The interview is part of a PhD project linked to Work Package 1 in NewTools. The PhD candidate is employed at OsloMet. The results will be published as a scientific article and in other forms of publications and presentations (scientific, public) and may also be used in teaching.

In this document, we provide you with information about the aims of the interviews and what participation will entail for you.

**Purpose**

The purpose of the study is twofold: 1) to investigate how you, as a project participant, experience participating in the interdisciplinary project NewTools, and 2) to investigate viewpoints on the two scoring systems to be developed.

**Who is responsible for the research project?**

OsloMet is responsible for the interviews (task 1.2 in the project description).

**Why are you being asked to participate?**

The invitation is extended to all project participants from both collaborating and associated partners (research institutions, government agencies, the food industry, and interest organizations). You are being asked to participate because you are a project participant and/or project manager for NewTools in your organization.

**What does participation entail for you?**

If you choose to participate in this study, you will be interviewed by the PhD candidate twice during the project period (spring/early summer 2022 and spring 2023). The interviews can be conducted digitally (via Zoom) or in person, at a time and place that suits you. We anticipate that the interviews will last approximately 45 minutes. The questions will revolve around two main themes: 1) the process to be carried out in NewTools (roles and engagement) and 2) viewpoints on the scoring systems. The first interview will focus on expectations for the process and viewpoints on the scoring systems. The second interview will primarily address experiences from the process so far. The interviews will be recorded digitally using a dictaphone app.

**Participation is voluntary**

Participation in the interviews is voluntary. If you choose to participate, you can withdraw your consent at any time without providing a reason by contacting us (see contact information below). All your personal data will then be deleted. There will be no negative consequences for you or your participation in NewTools if you choose not to participate or later decide to withdraw.

**Your privacy – how we store and use your information**

We will only use the information about you for the purposes described in this document. We treat the information confidentially and in accordance with data protection regulations.

**Who has access to your personal data?**

- Audio files from the interviews are only accessible to the PhD candidate and supervisors. The audio files cannot be listened to from the recording device. The files are stored and played in Nettskjema, where only the PhD candidate and supervisors have access through login and two-factor authentication.
- De-identified, transcribed interviews and analyses of the interviews are only accessible to the PhD candidate and supervisors at OsloMet. De-identified interviews are securely stored on OsloMet-Teams, which is access-controlled and protected with two-factor authentication.
- The PhD candidate will store the link between your name and contact details and the data material (audio files, Word files) in the form of a key code that is kept separate from other data.

**How will your answers be presented in publications?**

Your answers will be presented in relation to the sectors represented in NewTools: Research, government, the food industry, and interest organizations. We will not use the names of organizations or further specify the sector. Before publication, you will have the opportunity to review how your answers are presented by contacting the PhD candidate.

**What happens to your data when the research project ends?**

The data will be anonymized when the project concludes, which is planned for 2025. This will be done by destroying the code that links your name to the data material.

**What gives us the right to process your personal data?**

We process your data based on your consent.

On behalf of OsloMet, Sikt (Norwegian Agency for Shared Services in Education and Research) has assessed that the processing of personal data in this project complies with data protection regulations (reference number: 684528).

**Your Rights**

As long as you can be identified in the data material, you have the right to:

- Access the information we process about you and receive a copy of the data
- Correct any incorrect or misleading information about you
- Delete personal data about you
- Lodge a complaint with the Data Protection Authority regarding the processing of your personal data

**Contact Persons**

If you have any questions about the study, or if you wish to know more about or exercise your rights/withdraw from the study, please contact:

- OsloMet, Helen Engelstad Kvalem, heleneng@oslomet.no, tlf.: 900 40 589, or Anne Lene Løvhaug, annelene@oslomet.no, tlf.: 922 27 718.
- Data Protection Officer at OsloMet, Ingrid S. Jacobsen: personvernsombud@oslomet.no

If you have questions regarding the assessment of the project by Sikt, you can contact them via email (personverntjenester@sikt.no) or by phone: +47 53 21 15 00.

Kind regards,

Helen Engelstad Kvalem (researcher, supervisor)

Anne Lene Løvhaug (PhD student)
