# Supplementary material for: Norwegian food system actors’ perspectives on participating in a cross-sector research partnership: a qualitative study
Source: BMC Nutr. 2025 Dec 10;12:4. doi: 10.1186/s40795-025-01192-1 (PMC12784486; doi:10.1186/s40795-025-01192-1)
Supplement: Supplementary file 2 — Supplementary Material 2. [file 40795_2025_1192_MOESM2_ESM.docx]

*This interview guide has been translated from Norwegian to English for the purposes of this article.*

Interview guide NewTools – Expectations and experiences with project participation

**General introduction**

Introducing Anne Lene Løvhaug, involved in NewTools in Work Package 1 and 5. My role in the project involves partner involvement. I have been responsible for coordinating the work on the framework for engagement of partners. I will also be involved in the workshop process in Work Package 5.

The interviews are a sub-project aimed at investigating how partners experience participating in NewTools. The purpose is twofold:

- We want to understand what partners think about the profiling systems being developed. This is the core of the project. We know there are many different viewpoints on profiling systems for food in general, but we know less about opinions in Norway. Food profiling systems for nutrition and sustainability touch upon many different interests. We are particularly interested in where there are value conflicts – because they have a significant impact on the feasibility of implementing a scoring system.

- We also want to learn more about how partners experience participation in cross-sector partnerships within nutrition in general. The literature does not say much about this. In the short term, we are interested in following the partners in NT so that the project can be adjusted if necessary. In this sense, we are trying to understand how the framework for engagement will function. In the long term, we hope that experiences from NewTools can be used in future collaborative projects within nutrition by identifying facilitating factors.

**Information about data privacy**

Since the interviews are conducted by OsloMet, only I and my supervisors have access to the data from the interviews. All audio recordings and written documents are stored securely on OsloMet systems – you should feel confident that your responses are kept safe.

When presenting the results, we will associate responses from different participants with their respective sectors (research, food industry, government, interest organizations). We are aware that it is difficult to guarantee full anonymity in a project where the partners are publicly known. Therefore, we will consider how many quotes to use. Everyone will have the opportunity to review how their responses are presented before publication.

Is there anything you are wondering about before we proceed?

****START RECORDING HERE****

**Expectations for the Project**

- What considerations did your organization make regarding participation in the project?

- What expectations does your organization have for participation in and the results from the project?

- What benefits and drawbacks do you think there will be with this type of project collaboration for your organization?

**About the Process and Interaction in the Project**

- What do you think are the most important prerequisites for the collaboration between the different parties in the project to work?

- What is the most important thing for your organization that should be considered during the project period?

**Views on the food profiling systems**

Introduction:

The core of NewTools is the development of two profiling systems, one for nutritional quality and one for environmental and social sustainability.

NUTRITION:

The EU Commission has proposed the introduction of a harmonized system for nutritional quality across the EU. It has also been suggested that a new system could become mandatory.

- What do you think about a possible scoring system for nutritional quality in Norway?
- Follow-up: What is important to consider in a possible scoring system?
- What do you see as potential advantages and disadvantages of other food labeling schemes?

One of the systems considered relevant by the EU Commission, and which NewTools will base on, is called NutriScore.

- What are your organization's thoughts and considerations on the potential introduction of the NutriScore system in Norway?

SUSTAINABILITY:

In NewTools, a framework will be developed to assess both environmental and social sustainability of food products, which can form the basis for one or more scores.

- What do you think about a scoring system for sustainability for food products?
- Follow-up: What should be the key characteristics of a scoring system for sustainability for food products?
- What do you see as the benefits and challenges of developing a scoring system for sustainability for food products?

**Views on Roles and Interaction in the Project**

Introduction:

NewTools is a research project with partners across sectors, with many different interests and mandates. It resembles public-private partnerships. International literature on various partnerships within nutrition, criticizes public and private actors collaborating, based on the risk that conflicts of interest can undermine the results, especially relating toinitiatives for healthier food products.

- What do you think about this viewpoint in relation to NewTools?
- Follow-up: How do you envision handling potential conflicts of interest or value conflicts effectively?
- Are there any areas you think the project might face particular challenges?

The preliminary study and feedback on the framework indicate that it is important to have good and regular information and that partners have various opportunities to get involved, with sufficient time to provide input.

- How do you feel this has worked so far, at the start of the project?
- How do you expect to be involved moving forward?
- Is there anything else you would like to discuss?
